# The Course of Apparent Diffusion Coefficient Values following Perinatal Arterial Ischemic Stroke

**DOI:** 10.1371/journal.pone.0056784

**Published:** 2013-02-14

**Authors:** Niek E. van der Aa, Manon J. N. L. Benders, Koen L. Vincken, Floris Groenendaal, Linda S. de Vries

**Affiliations:** 1 Department of Neonatology, Wilhelmina Children's Hospital, University Medical Center Utrecht, Utrecht, The Netherlands; 2 Image Sciences Institute, University Medical Center, Utrecht, The Netherlands; Hôpital Robert Debré, France

## Abstract

**Background:**

Diffusion weighted MR imaging (DWI) plays an important role in the diagnosis of perinatal arterial ischemic stroke (PAIS) during the acute phase. Its derived apparent diffusion coefficient (ADC) can be used to quantify the diffusion restriction. Aim of the current study was to identify the changes in ADC values in the acute phase following PAIS.

**Methods:**

A cohort of 36 infants with a confirmed PAIS who were examined once during the first ten days of life was studied. ADC values in the core of the ischemic tissue (iADC) were determined and correlated with postnatal age. ADC ratios (rADC) were calculated by dividing the iADC value by the ADC value of the corresponding area in the contralateral ‘healthy’ hemisphere.

**Results:**

Infants were scanned between days two and ten. A non-linear increase in iADC and rADC values was observed over time and large middle cerebral artery strokes resulted in lower iADC and rADC values. Normalisation of rADC values was observed after day seven. rADC values were lower when compared to previously published rADC values of infants with hypoxic ischemic encephalopathy, suggesting more severe injury.

**Conclusions:**

Following PAIS, DWI showed decreased ADC values with a non-linear increase during the first week, and pseudonormalization after day 7, which limits the use of DWI to assess PAIS to the first week. Compared to previous studies, ADC values were lower when compared to infants with hypoxic ischemic encephalopathy, most likely due to more severe injury.

## Introduction

Perinatal arterial ischemic stroke (PAIS) is a serious condition with an incidence of 1∶2300–4000 newborns and often results in long-standing neurodisabilities [1], [2]. Approximately 70% of all infants present with (hemi)convulsions, initiating the diagnostic process [3]. Clinical symptoms can however also be more subtle and non-specific, including irritability, hypotonia and apnea [1], [4].

For the eventual diagnosis MRI is superior to cranial ultrasound or CT [5]. Several MRI sequences can be used for the diagnosis of PAIS, including T1, T2 and diffusion weighted imaging (DWI), MR spectroscopy and MR angiography. DWI plays an important role in the diagnosis of PAIS because of its high sensitivity for detection of ischemic lesions in the acute phase. The rapid shift of water from the extracellular space to the intracellular space, resulting in cytotoxic edema, can be assessed within hours as increased signal intensity (SI) on DWI and as decreased SI on the derived apparent diffusion coefficient (ADC) map, preceding the signs of ischemia on conventional MRI [6]. Moreover it can be used to predict the development of hemiplegia based on involvement of the corticospinal tracts, which is often difficult to see shortly after the stroke on conventional T1 and T2 weighted imaging [7]–[9].

The time course of ADC values has been previously studied and documented in newborns with hypoxic-ischemic perinatal brain injury [10]–[13]. These studies mainly included infants with hypoxic ischemic encephalopathy (HIE) following perinatal asphyxia. Lowest ADC values have been observed between days two and four, after which ADC values increase again. At approximately six to eight days after the injury, ADC values in the ischemic tissue appear to have normalized, i.e. the so-called pseudonormalization, at which point in time DWI has become of limited value in diagnosing PAIS [10]–[13]. T2-weighted imaging will now clearly show abnormal signal intensities in the ischemic tissue [14]. Following this period, there is a further increase of the ADC during the subsequent days reflecting the underlying cell lysis and vasogenic edema which will eventually result in cystic evolution.

Little is known about changes in ADC values following PAIS. By using a visual SI classification Dudink et al showed changes on DWI following PAIS with a similar course as described in infants with HIE [14]. They did not, however, quantify the changes over time. Aim of the present study therefore was to examine and quantify the course of ADC values following PAIS in full-term infants.

## Materials and Methods

### Patients

Full-term infants born between 2003 and 2011 with a confirmed PAIS on MR imaging after admission to our level III neonatal intensive care unit at the Wilhelmina Children's Hospital were included in this study. Infants were admitted because of HIE or (sub)clinical seizures and MRI of the brain was performed for clinical reasons. Clinical and obstetric characteristics were collected from the infants' charts, including the time of birth. If the first clinical presentation was beyond 48 hours, infants were not included as a perinatal onset was less likely in these infants. This included infants with a sepsis, infants with focal ischemia due to congenital heart disease and infants with a stroke secondary to an intracranial haemorrhage.

Informed verbal parental consent was obtained to perform the MRI for clinical purpose. Due to the retrospective design of this study, no written parental consent was acquired for the use of MRI data in this study. The institutional review board of the University Medical Center Utrecht did however approve the use of MRI data for anonymous data analysis and waived the requirement to obtain informed consent.

### Imaging methods

MRI was performed on a 1.5T Phillips Gyroscan ACS-NT or Achieva MR system (Philips Medical Systems, Best, Netherlands). Axial images were acquired with a 128×77 matrix (256×256 reconstruction matrix) and a field of view 180×180 mm. DWI was acquired in 3 orthogonal directions using single-shot echo planar imaging resulting in 25 slices with a 4 mm thickness (TR 3700–5200 ms, TE 89 ms, b values of 0 and 1000 mm^2^/s, no gap). An ADC map was created at the MR console and was used for further analyses.

The protocol further included an axial turbo spin echo T2 (TR/TE 7600/150) and an axial turbo spin echo inversion recovery (TR/TI/TE 4000/600/30) sequence. A second scan was acquired using the same protocol at the age of three months to evaluate the residual damage.

Infants were sedated throughout the examination with a combination of pethidine (2 mg/kg body weight), chlorpromazine (0.5 mg/kg body weight) and promethazine (0.5 mg/kg). Infants who required mechanical ventilation were sedated with morphine (0.1 mg/kg i.v.) followed by administration of 0.1 mg/kg i.v. of vencuronium, to avoid movement artefacts. A vacuum pillow (Med-Tec, Orange City, IA) was used to prevent movement of the head and Minimuffs® (Natus Medical Inc, San Carlos, CA) were used for hearing protection. Intensive care was continued throughout the examination with the attendance of a neonatologist, and the heart rate and transcutaneous oxygen saturation were monitored by pulse oximetry in all infants (Nonin, Minneapolis, MN), as well as respiration rate (Philips ACS-NT, Best, The Netherlands).

### Image analysis

**Figure 1:**
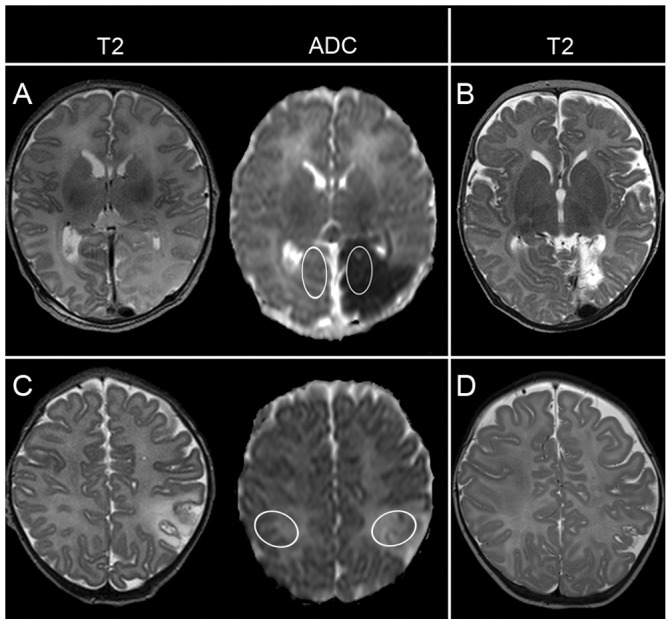
Examples of ADC measurements in the neonatal ADC map. This infant was scanned on day 4 and suffered a posterior cerebral artery stroke (A). The ROI is placed in the region that corresponds with the cystic evolution at three months (B) and a corresponding ROI is placed in the contralesional hemisphere. The same is shown for an infant with a cortical MCA stroke who was scanned on day 10 (C), but shows focal atrophy at three months. (D).

The ADC maps and conventional images were digitally transferred to a standalone Philips workstation. The ADC value in the ischemic tissue (iADC) was determined by means of manually drawn ellipsoids in the core of the stroke, carefully avoiding the inclusion of any CSF, which would increase the mean iADC value. As the size and visibility of the stroke on the ADC map changes over time, we tried to standardize the placement of the ROI. This was done by determining the region with most tissue loss on a second scan, acquired at the age of three months, usually seen as an area of cavitation. This region was considered to be the core of the stroke, rather than the penumbra, and the ROI was placed in the corresponding region on the neonatal ADC map (Figure 1).

As the sizes of the strokes varied, the size of the ROI also varied, with smaller ROIs in smaller strokes. To reduce the partial volume effect, ROIs were only drawn if the ischemic tissue was visible on at least three consecutive slices. If the ischemic tissue extended over more than three slices, or in case of multiple ischemic lesions, multiple ROIs were used and averaged.

Additional ROIs of the same size were placed in the corresponding regions of the contralateral hemisphere. These were used to calculate an ADC ratio (rADC) by dividing the iADC values by the ADC value of the corresponding ROIs of the contralateral hemisphere. All analyses were performed twice by the same observer (NvdA) to calculate the intra-observer reproducibility.

### Clinical follow-up

All children were seen at regular intervals in the neonatal follow-up clinic. Gross motor development was assessed with items from Amiel-Tison and Touwen [15], [16]. The Griffiths' mental developmental scale (GMDS) was used to obtain a developmental quotient at 18 to 24 months [17]. Presence of a hemiplegia was assessed according to Claeys et al [18].

### Statistical analysis

The Kruskal-Wallis test or Fisher's exact test was used to analyse any differences in clinical characteristics. To assess the relationship between the postnatal age and the ADC values, linear and quadratic regression analysis was performed with Prism 5.0 (Graphpad, La Jolla, CA). Results of the models were compared using the Akaike's information criterion (AIC) where a lower value indicates a better fit. The relation between ADC values and clinical outcome was assessed with linear or logistic regression where appropriate. SPSS v19 (SPSS Inc. Chigaco, IL.) was used to perform regression analyses and to calculate the intra-observer reproducibility.

## Results

We included 36 infants who were admitted during their first week after birth (Table 1). Clinical or subclinical seizures were observed in 35 infants. A median number of three anti-epileptic drugs was required (range 1–5) and most seizures (27/35 infants, 54%) responded to therapy within the first 72 h after birth. The remaining infant presented with perinatal asphyxia. A stroke was most frequently observed in the territory of the middle cerebral artery (MCA, n = 32, 89%). The remaining four infants suffered a stroke in the posterior cerebral artery territory. No differences in postnatal age at scan were found between the different stroke subtypes. Six infants (17%) also showed an ischemic area, contralateral to the stroke. In one infant, the contralateral ischemia was so extensive that no rADC was calculated.

**Table 1:** Clinical characteristics of studied infants reported as median and [range].

	MCA					PCA	Significance
	Main trunk	Posterior trunk	Anterior trunk	Lenticulostriate branches	Cortical branch		
Gender (m/f)	7/2	5/7	1/1	0/1	5/3	3/1	n.s.
Gestational age (w)	40^+4^ [37^+6^–42^+1^]	40^+6^ [37^+2^–41^+5^]	41^+2^ [40^+4^–42^+1^]	37^+5^	38^+6^ [37^+1^–42^+5^]	40^+2^ [39^+0^–41^+3^)]	n.s.
Birth weight (g)	3250 [2600–4360]	3390 [2150–3860]	3521 [3470–3572]	2305	3120 [2740–3530]	3650 [2145–3880]	n.s.
Apgar score 1′	6 [2–9]	9 [1–9]	8 [6–9]	6	5 [1–9]	3 [1–5]	n.s.
Apgar score 5′	7 [6–10]	9 [4–10]	9 [9–10]	7	8 [4–10]	8 [7–8]	n.s.
Instrumental delivery	6	7	1	-	6	2	n.s.
Seizures	9	12	2	1	8	3	n.s.
*Duration>72 h*	5	3	0	0	0	0	n.s.
*Anti-Epileptic drugs*	3 [2–4]	3 [1–5]	1	1	2 [1–5]	1 [1–4]	n.s.
Asphyxiated	1	3	0	1	2	1	n.s.
Follow-up (n)	8	12	0	1	7	4	
Hemiplegia	8	1	-	0	0	0	*p<0.001* ¶
Griffiths' DQ	100 [55–105]	104 [97–126]	-	96	106 [83–123]	102 [92–121]	n.s.

In infants with (sub)clinical seizures, a distinction was made between seizures that responded to anti epileptic drugs within the first 48 h after birth and seizures that required treatment beyond 72 h. Follow-up data, including the Griffiths' Developmental Quotient (DQ) are given for those infants who were tested between the age of 18 and 24 months.

¶ Main trunk MCA vs other strokes.

The second scan, acquired at the age of three months, showed cystic evolution in 25 infants (69%). On the 11 remaining MRI's only local atrophy could be observed in the prior stroke area.

**Figure 2:**
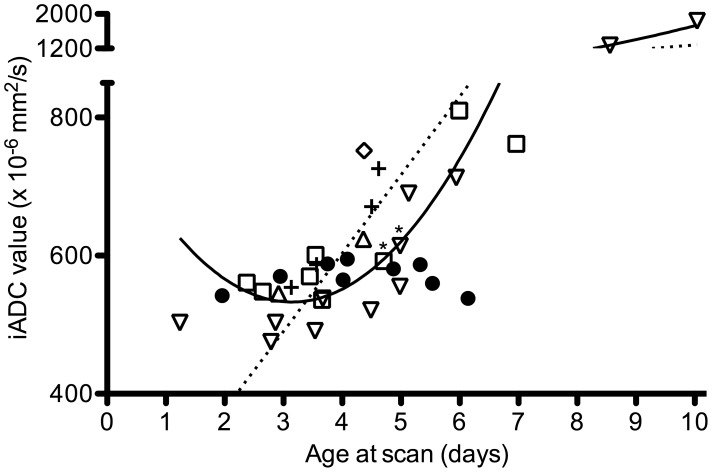
Time course of the ADC values. ADC values were determined in the core of the stroke in 36 infants with a MCA main trunk (•), anterior MCA trunk (▵), posterior MCA trunk (▿), lenticulostriate branches (⋄), cortical branch (□) and PCA (+) stroke. The regression lines for the linear (dashed line) and quadratic regression (continuous line) are depicted. Two infants who were scanned following total body cooling are identified with an asterisk (*).

**Figure 3:**
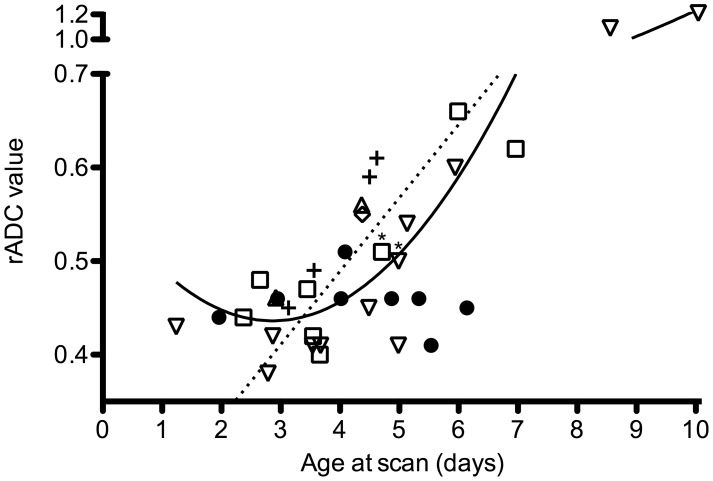
Time course of the ADC ratio. ADC ratios were determined in 35 infants with a MCA main trunk (•), anterior MCA trunk (▵), posterior MCA trunk (▿), lenticulostriate branches (⋄), cortical branch (□) and PCA (+) stroke. One infant with extensive bilateral ischemia was not included. The regression lines for the linear (dashed line) and quadratic regression (continuous line) are shown. Two infants who were scanned following total body cooling are identified with an asterisk (*).

Both iADC and rADC values could be reproduced accurately with an intraclass correlation coefficient of .90 and .91 respectively. When linear and quadratic regression analyses were performed (dependent: iADC, independent: postnatal age), the quadratic analysis resulted in a better fitting model (R^2^ = 0.90 vs R^2^ = 0.64, Figure 2), reflected by a lower AIC. The same was found for the rADC (R^2^ = 0.86 vs R^2^ = 0.64, Figure 3). All infants scanned up to day seven showed a rADC below 0.7 and only the two infants scanned on day nine and ten showed a rADC above 1. No correlation was found between the ADC value in the unaffected hemisphere and the postnatal age.

Most infants were scanned between days three and seven (n = 32, 89%). During this period, linear regression analysis resulted in a better fitting compared to quadratic regression analysis. We therefore applied multiple linear regression to the data acquired in this period. As it was our impression from clinical experience that large MCA strokes show low ADC values during a longer period, the presence of a large MCA stroke, defined as the presence of a main trunk or posterior trunk MCA stroke, was included in the analysis (dependent: iADC/rADC, independent postnatal age, presence large stroke). Besides the postnatal age, the presence of a large MCA stroke was indeed found to be associated with both the iADC and rADC values. For the iADC, adding the presence of a large MCA stroke improved the model (R^2^ .56 vs. R^2^ .39). The presence of a large stroke resulted in a decrease in iADC value of 69 (95% C.I. −111–−27). The same was found for the rADC (R^2^.54 vs .31), with a decrease in rADC of .07 (95% C.I. −.11–−.03). To study whether the course of ADC values over time were different in large strokes, we calculated an interaction between postnatal age and presence of a large stroke. This did however not contribute to the model, suggesting that the slope of the linear regression line (i.e. the speed at which the ADC value normalized) was not different between both groups. Finally, no association was found between the pattern of injury observed at three months (a cyst or focal atrophy) and the neonatal iADC or rADC values.

Nine children were diagnosed with a hemiplegia at follow-up, which was more frequently observed in infants with a main branch MCA stroke (*p*<0.001, Table 1). No difference in iADC or rADC values was observed between the children who did and did not develop a hemiplegia. Forward logistic regression showed that only presence of a large stroke was predictive of development of a hemiplegia. No association was found between any variable and the Griffiths' developmental quotient.

## Discussion

In the current study we have been able to demonstrate a positive correlation between the postnatal age and the ADC values in the stroke area in 36 infants with PAIS. The etiology of PAIS is multifactorial and includes maternal, placental, coagulation, cardiac and infectious disorders [19]. By carefully selecting only infants with early onset of symptoms, i.e. within 48 hours after birth, we tried to create a homogeneous group in which stroke likely occurred in the immediate perinatal period [20]. This is the first study describing the changes in ADC values following PAIS in such a large cohort. Our finding has important implications for the use of DWI in the acute phase, as it only shows low ADC values during the first week. DWI after the first week therefore seems to be of limited value and conventional imaging will then be required to diagnose PAIS. Not performing DWI during the first week in infants suspected of PAIS should therefore be regarded as a missed opportunity. However, in some cases, e.g. in unstable infants, MRI during the first week may not be possible. In these cases, signal intensity changes on T1- and T2-weighted images can be used for a prolonged time period to identify stroke areas [14].

Besides the diagnosis of ischemic injury, DWI can also be used to predict motor outcome following PAIS [7]–[9]. Involvement of the corticospinal tracts on DWI is predictive of motor disabilities and is referred to as ‘pre-Wallerian degeneration’. Our study does not suggest any additional value of a low ADC value by itself for prediction of motor outcome. Low ADC values were found in both small strokes, resulting in focal atrophy, and in larger strokes, resulting in cysts. The size of the lesion rather than the ADC value itself was predictive whether the stroke would evolve into an area of cavitation or only atrophy. The size and especially the site of the area of infarction will eventually determine whether a motor handicap will occur [21]. Furthermore involvement of the cerebral peduncle has been found to be an important predictor [9], [22]. While we did not report any data on the descending corticospinal tracts at the level of the cerebral peduncles, Husson et al. recently reported DWI findings in a cohort of 73 infants with PAIS and noted that DWI may not yet show any abnormalities in the corticospinal trajectory on day two [9]. Combined with our findings, this suggests that the time window for optimal usage of DWI is between days three and seven.

Few other studies have reported DWI changes over time following PAIS. Cowan et al. were the first to report the use of DWI following PAIS and found a high SI during the first week and normal SI from day seven onwards [23]. Similar findings were reported by other studies [24]–[26]. In a more recent study, Dudink et al. used a visual SI score to quantify the changes in SI on DWI over time and found the highest score from day two to five, though in some cases a high SI was observed until day 12. Our data suggest that the diffusion abnormalities resolve around day eight or nine, though an accurate estimation is difficult as few infants were scanned beyond the first week. This difference might be explained by the more confined inclusion criteria used in our study, requiring an onset of symptoms within 48 hours. Furthermore, the prolonged period during which high signal intensities were observed might also be explained by the visual classification used by Dudink et al, which gives a broader visual gestalt, than the focal measurement of ADC values.

The time course described here shows a similar pattern as the time course described by McKinstry et al. who studied serially obtained ADC values of ten infants with a brief hypoxic ischemic perinatal event resulting in HIE [10]. MRI revealed a spectrum of injury, including injury to the BGT and watershed areas. McKinstry also calculated rADC values, but used a group of healthy controls as a reference. They observed a nadir at day 2–3, with a rADC of 0.65 and normalization from day seven onwards (Table 2). When we compare the courses of rADC values, we can observe that the rADC values following PAIS tend to be lower between day two and day eight. This suggests more severe injury, reflected by an increase in cytotoxic edema and often followed by cystic evolution. This difference might be explained by the underlying mechanism. In the McKinstry study, the adverse perinatal event was brief. Even though the exact mechanism of PAIS is still to be revealed, the main hypothesis is a cerebral thrombo-embolism causing a temporary or permanent cerebral arterial occlusion resulting in prolonged ischemia and more severe injury [19]. In a number of animal models, permanent or prolonged time periods of ischemia resulted in lower ADC values [27]–[30]. Lower ADC values are also associated with tissue loss, as shown in an animal model of cerebral ischemia, where areas of low ADC values showed no restoration of ADC values following reperfusion and resulted in focal infarction on subsequent histopathology [31]. Likewise in adults, rADC values are lower following a stroke when compared to a transient ischemic attack [32] and are higher in stroke areas with restored perfusion [33]. Similar findings have been reported in infants with HIE following perinatal asphyxia, in whom lower ADC values were associated with focal infarction [11]. More recently, Bednarek et al. showed that in infants who underwent therapeutic cooling following HIE, the largest reduction in ADC was observed in infants with the most severe injury (Table 2) [34].

**Table 2:** Median rADC values of infants with HIE as reported by McKinstry et al. [10] and Bednarek et al. [34].

	Age at MRI (days)						
	1–2	3	4	5	6	7	8–10
HIE, McKinstry et al. (n = 10)	0.84	0.58	0.71	0.94	0.87	0.93	0.94
HIE, Bednarek et al. (n = 23)							
*Mild/moderate*	0.75	0.75	0,70	0.72	0.76	0.8	0.81
*Severe*	0.48	0.52	n.d.	0.35	n.d.	0.58	0.76
PAIS, current study (n = 36)	0.43	0.45	0.43	0.51	0.54	0.54	1.1

Both studies performed serial measurements, which were compared to ADC values of healthy controls. In the study of Bednarek, infants received therapeutic hypothermia. Based on MRI, the patterns of injury were classified as mild/moderate or severe. On some days no data (n.d.) were available.

Following both experimental stroke and human stroke, lowest ADC values have been observed in the ischemic core, with a gradient of higher ADC values toward the border of the lesion [35], [36]. Serial measurements in rats have shown that if the blood flow is not restored, the core of the lesion continuously spreads to surrounding areas of moderately reduced ADC values [36]. In a recent study in infants with PAIS studied with perfusion weighted imaging (PWI), a similar gradient of blood perfusion of the stroke area was observed with decreased perfusion in the core and in increased perfusion in the periphery of the stroke [37]. This may also explain why ADC values were found to be lower in large MCA strokes. It is likely that part of the periphery of the stroke, with restored blood flow and therefore higher ADC values, was included in the ROI in the smaller strokes, resulting in a higher ADC value. We were however unable to study this, as PWI was not performed in this group.

In our study pseudonormalization occurred around days eight and nine. In previous studies reporting changes in ADC following perinatal asphyxia this occurred from day seven onwards, though some infants already showed near normal rADC values before day seven [10]–[13]. In animal studies no significant differences have been observed between the different durations of ischemia and the time to pseudonormalization [30]. Only following permanent occlusion, pseudonormalization has been shown to occur later [29]. A possible explanation for the slightly later pseudonormalization observed in our cohort might be a delayed restoration of blood flow which has been observed following PAIS [30].

In animal models, brief periods of ischemia result in a decline of ADC values within minutes. After reperfusion, the ADC values rapidly restore to normal to show a secondary ADC decline after 6–48 hours, depending on the type of animal [28], [38]. Following prolonged or permanent ischemia, no restoration of ADC values is observed and ADC values remain low until pseudonormalization occurs [29], [30]. We were unable to identify the ADC changes during the first two days, as only two infants were scanned before day three. It would be of interest, however, to scan infants sequentially during the first days of life. This may differentiate between this secondary ADC decline and permanent low ADC value, thereby unraveling part of the pathophysiological mechanism of PAIS.

Two infants with HIE received body cooling during 72 h and were both scanned on day five after birth. Selective head or whole body cooling interrupts the complex cascade following HIE leading to neuronal cell death [39]. Recent data suggest that cooling results in delayed pseudonormalization, though this has not been studied in infants with PAIS [34]. As the ADC values of the two cooled infants in our cohort did not differ from the other infants, they were not excluded.

There are some limitations to this study. One is the limited number of scans before day three and beyond day seven due to the retrospective design of this study. Many of the included infants were referred to our hospital and were not admitted and therefore not scanned before day three. Scanning beyond day seven is rarely performed as we try to obtain a diagnosis as soon as possible in infants with seizures of unknown origin. To obtain more information about the changes in ADC values, a prospective, longitudinal study would be required to acquire sequential ADC values in the same patient over time. At present, the intensive care of these infants precluded repetitive measurements during the first week. Yet, despite this limitation, we were able to show lower iADC values in large MCA strokes and show lower rADC values when compared to the McKinstry study.

Second, one should be cautious when comparing ADC values acquired with different scanners or scanning parameters [40]. Yet, any differences in ADC values due to scanner differences are likely to be overcome by using a ratio, as done in this study. This also allowed us to correct for any differences in ADC values due to gestational age or affected structures, as both are known to influence the ADC value [11], [41]. This rADC however, requires an unaffected, contralesional hemisphere. Six infants also showed ischemic lesions in the contralateral hemisphere. In most cases these were small isolated ischemic lesions, but in one infant the injury was so extensive that calculation of rADC was not informative. Furthermore it is known that transsynaptic degeneration can also cause early changes in ADC values in non-primary involved areas [42]. This might be solved by the use of controls, but at present ethical considerations prevent the MRI examination of healthy neonates in our country. However, if the ADC values in the contralateral hemisphere would indeed be lower, usage of ADC values of healthy controls would result in even lower rADC values, thereby strengthening the difference between the course described by McKinstry and the rADC values as described in this study.

A third limitation is the fact that exact timing of PAIS was not possible. Timing of neonatal brain injury, including PAIS, has been a subject of debate for a long time, which is also due to its multifactorial pathogenesis [19]. Recent evidence however, especially MRI data, indicates that brain injury mainly occurs in the immediate perinatal period [14], [43]. After carefully selecting our included patients, our findings are in line with these data, as the homogeneous time course observed suggests that the onset of the injury was similar for all infants. Furthermore, all infants showed clinical signs within 48 hours after birth, suggestive of an immediate perinatal onset [14].

In conclusion, following PAIS, a non-linear increase in ADC values can be observed during the first week, with pseudonormalization after day 7, limiting the use of DWI to detect PAIS to the first week. Compared to a previous study in infants with HIE, these ADC values seemed lower, most likely corresponding to more severe injury.
